# The Prevalence of Sexually Transmitted Infections in Papua New Guinea: A Systematic Review and Meta-Analysis

**DOI:** 10.1371/journal.pone.0015586

**Published:** 2010-12-23

**Authors:** Andrew Vallely, Andrew Page, Shannon Dias, Peter Siba, Tony Lupiwa, Greg Law, John Millan, David P. Wilson, John M. Murray, Michael Toole, John M. Kaldor

**Affiliations:** 1 National Centre in HIV Epidemiology and Clinical Research, University of New South Wales, Sydney, Australia; 2 School of Population Health, University of Queensland, Herston, Brisbane, Australia; 3 Papua New Guinea Institute of Medical Research, Goroka, Papua New Guinea; 4 National AIDS Council Secretariat, Port Moresby, Papua New Guinea; 5 National Department of Health, Port Moresby, Papua New Guinea; 6 School of Mathematics and Statistics, University of New South Wales, Sydney, Australia; 7 Centre for International Health, Burnet Institute, Melbourne, Australia; Yale University School of Medicine, United States of America

## Abstract

**Background:**

The potential for an expanded HIV epidemic in Papua New Guinea (PNG) demands an effective, evidence-based and locally-appropriate national response. As sexually transmitted infections (STIs) may be important co-factors in HIV transmission nationally, it is timely to conduct a systematic review of STI prevalences to inform national policy on sexual health and HIV/STI prevention.

**Methodology/Principal Findings:**

We undertook a systematic review and meta-analysis of HIV and STI prevalences in PNG, reported in peer-reviewed and non-peer-reviewed publications for the period 1950–2010. Prevalence estimates were stratified by study site (community or clinic-based), geographic area and socio-demographic characteristics. The search strategy identified 105 reports, of which 25 studies (10 community-based; 10 clinic-based; and 5 among self-identified female sex workers) reported STI prevalences and were included in the systematic review. High prevalences of chlamydia, gonorrhoea, syphilis and trichomonas were reported in all settings, particularly among female sex workers, where pooled estimates of 26.1%, 33.6%, 33.1% and 39.3% respectively were observed. Pooled HIV prevalence in community-based studies was 1.8% (95% CI:1.2–2.4) in men; 2.6% (95% CI:1.7–3.5) in women; and 11.8% (95% CI:5.8–17.7) among female sex workers.

**Conclusions/Significance:**

The epidemiology of STIs and HIV in PNG shows considerable heterogeneity by geographical setting and sexual risk group. Prevalences from community-based studies in PNG were higher than in many other countries in the Asia-Pacific. A renewed focus on national STI/HIV surveillance priorities and systems for routine and periodic data collection will be essential to building effective culturally-relevant behavioural and biomedical STI/HIV prevention programs in PNG.

## Introduction

Recent published estimates suggest Papua New Guinea (PNG) has among the highest adult HIV prevalences in the Asia-Pacific region [1], [2], [3], [4], [5], estimated at 1.28% among people aged 15–49 years in 2007[2], although more recent estimates suggest national prevalence may be closer to 1.0%[6]. The epidemic is primarily linked to heterosexual transmission[1], [5], [7], [8] and exhibits substantial geographic heterogeneity, with over half the reported HIV diagnoses coming from the capital, Port Moresby; 20% from Western Highlands; and 10% from Morobe Province[2]. Modelling projections suggest that by 2025, adult HIV prevalence could be as high as 10% and around 300,000–400,000 people will have died from AIDS-related illness[9], [10]. Innovative strategies for HIV prevention, treatment and care are urgently needed to address this complex public health issue in a country with unparalleled geographical, linguistic and cultural diversity[5], [11]. To support the planning and evaluation of these strategies, surveillance systems must be capable of monitoring behavioural and biomedical trends over time in key populations[5], [8].

In addition to monitoring HIV, it is important to be able to track other STIs. These infections indicate the presence of HIV-related risk behaviour, as well as biologically enhancing the transmission and acquisition of HIV itself[12], [13], [14], [15], [16], [17], [18], [19]. Their effective management may play an important role in HIV prevention[20], [21], [22], [23], [24]. Like many countries, PNG could benefit from better information on the extent of STIs to inform prevention and control strategies. This information would also facilitate the development of locally-relevant mathematical models that could be used to estimate the likely progression and impact of HIV and STI epidemics under different policy scenarios.

In this paper we present the first systematic review and meta-analysis of the epidemiology of STIs, HIV and genital infections in PNG, and discuss the implications of these findings for public health policy.

## Methods

### Literature Searches

A systematic electronic search of Embase (1950 to May 2010), Medline (1966 to May 2010) and Pub Med Central (1951 to May 2010) databases was conducted to identify published peer-reviewed studies using the following Medical Subject Headings (MeSH) terms: ‘sexually transmitted infections’, ‘sexually transmitted diseases’ or ‘venereal diseases’ and ‘Papua New Guinea’. Other keywords were included for each STI of interest: ‘HIV’, ‘herpes’, ‘HSV-2’, ‘*Chlamydia trachomatis*’, *‘Treponema pallidum’*, ‘*Trichomonas vaginalis*’, ‘*Neisseria gonorrhoeae’*, ‘bacterial vaginosis’, ‘chancroid’, ‘donovanosis’, ‘lymphogranuloma venereum’, ‘LGV’ and ‘syphilis’. The search strategy also included the use of truncated and wild card forms of the above, and secondary searching of the reference lists of studies located in the above databases. No language restrictions were applied. All unpublished abstracts (from conference proceedings and internal reports) held in the PNG Institute of Medical Research (PNGIMR) library in Goroka, were hand-searched, as was the PNG Medical Journal dated from 1996–2007. Unpublished reports from the National Department of Health and the National AIDS Council were also located where available. Three reviewers (AV, AP, SD) independently screened potential publication titles and abstracts, and examined full text manuscripts.

### Selection of Studies

Sources of selection and measurement bias were considered in the inclusion and exclusion of studies in terms of study design and context, outcome measure, and participants. Measurement bias was addressed by only including studies that reported laboratory-confirmed estimates and excluding those where estimates were based on self-reported or clinical diagnosis. Studies were included if they reported either the prevalence or incidence of at least one STI pathogen, separately for males and females, and where STI diagnosis was confirmed by a laboratory assay. We only considered studies based on individual-level study designs, including cross-sectional prevalence surveys, longitudinal cohort studies, and studies based on hospital or STI clinic presentations.

The likely effect of selection bias on reported STI prevalence was also considered, and studies were categorised as being conducted in general populations, clinic populations, and in specific sub-groups potentially at increased risk of HIV/STI acquisition (e.g. women who engage in commercial or transactional sex work). We excluded commentaries, narrative reviews, case series, qualitative and anthropological studies, and studies reporting prevalence estimates in children less than 10 years.

### Definition of Outcomes

The primary outcome was laboratory-confirmed HIV/STI incidence or prevalence as defined by the authors of each study. Studies reporting prevalences based on clinical criteria alone were not considered to have met the outcome definition and were excluded from this review.

### Data Extraction and Data Analysis

From each report, data were extracted on study design, setting (community-based or at-risk populations), and geographical location (urban/rural; village/town/province). Separate tabulations were prepared for each STI, and prevalence estimates extracted by sex, age-group, study-setting and location. Pooled prevalence estimates and 95% confidence intervals, stratified by study-setting and gender, were determined by random effects meta-analysis using the inverse variance method (DerSimonian-Laird)[25], with between study heterogeneity assessed using the *I^2^* statistic. Where available, adjusted prevalence estimates (e.g. by age) were used in the meta-analysis. If adjusted estimated were not reported, unadjusted estimates and exact 95% confidence intervals were calculated from extracted data. Meta-analysis was carried out in Stata Version 10.1 (Stata Corp LP, Texas, USA).

## Results

**Figure 1:**
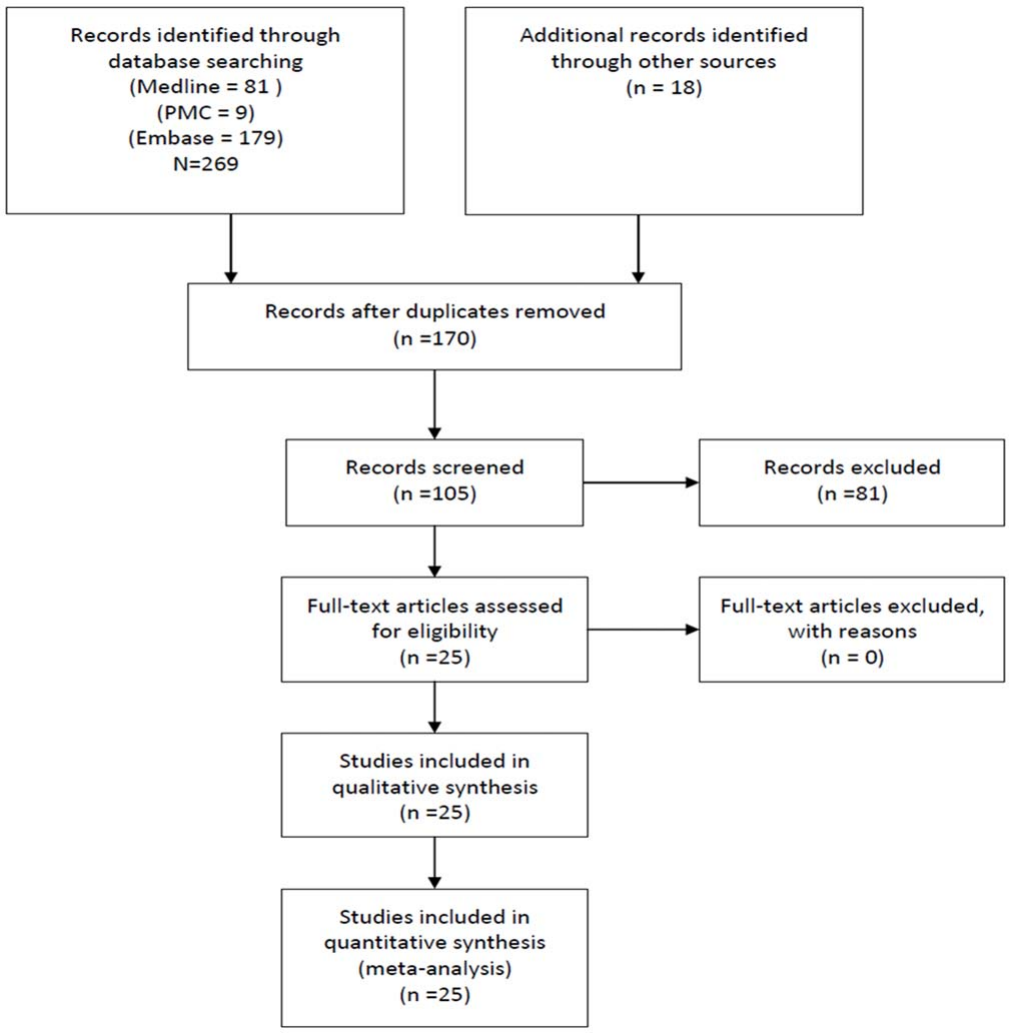
Flow chart of identification and selection of studies for inclusion.

The above search strategy resulted in 105 reports, including 25 studies that reported STI prevalence estimates (10 community-based studies; 10 clinic-based studies; and 5 studies among female sex-workers (FSWs) recruited via community outreach), which were included in this review (Figure 1; Table S1). Of these 25 studies, 16 were conducted in the period 1995–2000; 5 between 2001–2005; and 2 between 2006–2010. Two earlier studies (1994; 1977) were also included in the review.

Fourteen studies reported on *Chlamydia* [26], [27], [28], [29], [30], [31], [32], [33], [34], [35], [36], [37], [38], [39] and *Trichomonas* [27], [29], [31], [32], [33], [35], [37], [38], [39], [40], [41], [42], [43], [44]; 11 on gonorrhoea [26], [27], [28], [29], [31], [32], [33], [34], [37], [38], [39]; 10 on syphilis [26], [27], [28], [29], [33], [37], [38], [39], [45], [46]; 9 on HIV [26], [27], [29], [33], [39], [45], [46], [47], [48]; 2 on HSV-2 prevalence [49], [50]; and 3 on bacterial vaginosis [37], [38], [43]. We were unable to identify laboratory-confirmed prevalence estimates for lymphogranuloma venereum or chancroid [51], [52], [53], [54], [55], [56] and identified only one study reporting donovanosis (among 14/210 (6.7%) men and 5/64 (7.8%) women attending STI clinics in five urban centres[28]). No published or unpublished prevalence estimates among men that have sex with men (MSM) in PNG were identified in this systematic review.

**Figure 2:**
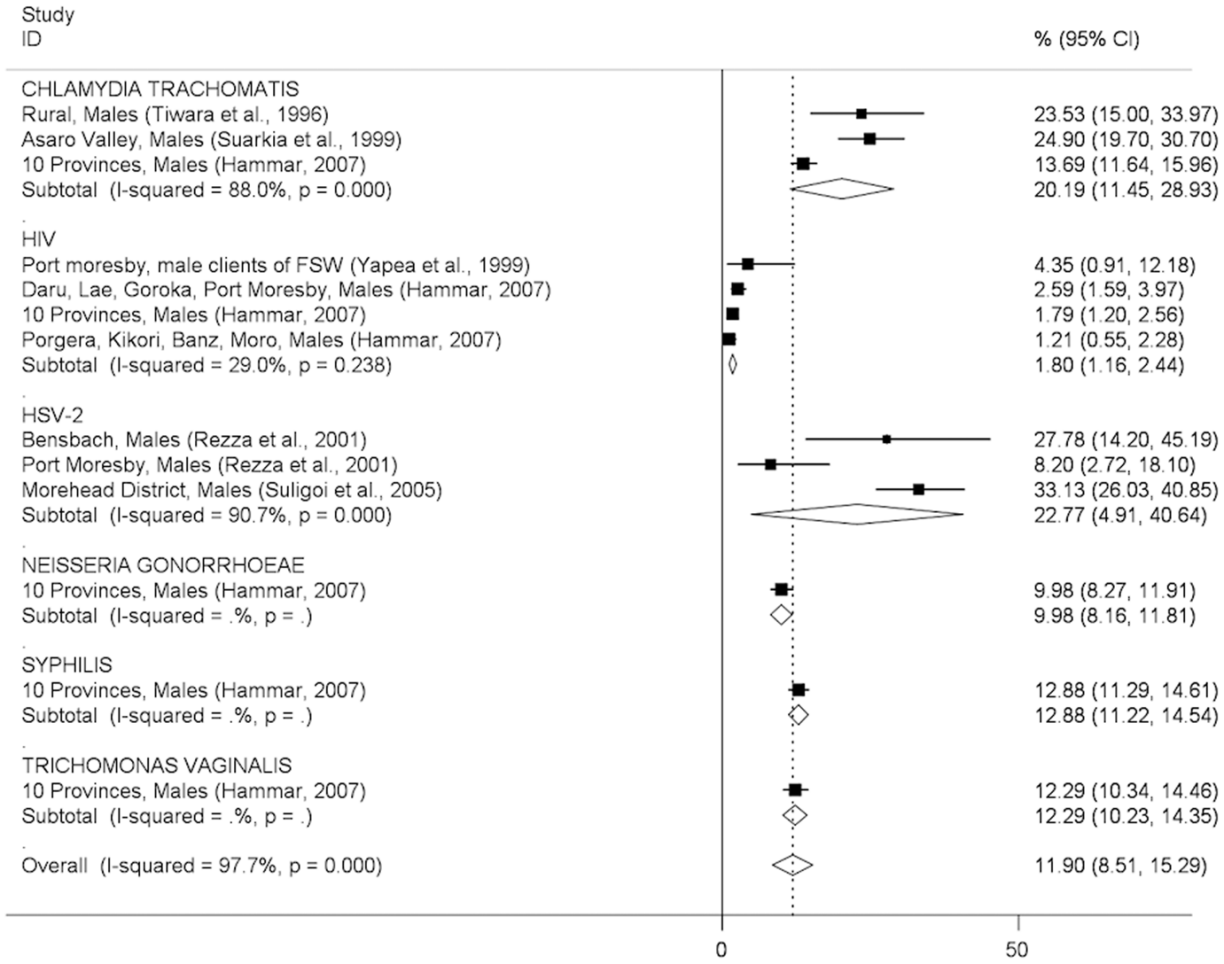
Prevalence of sexually transmitted infections in men: community-based studies. Forest plots showing unadjusted prevalence estimates (boxes) with 95% confidence limits (bars). Pooled prevalence estimates are represented as diamonds in this plot.

**Figure 3:**
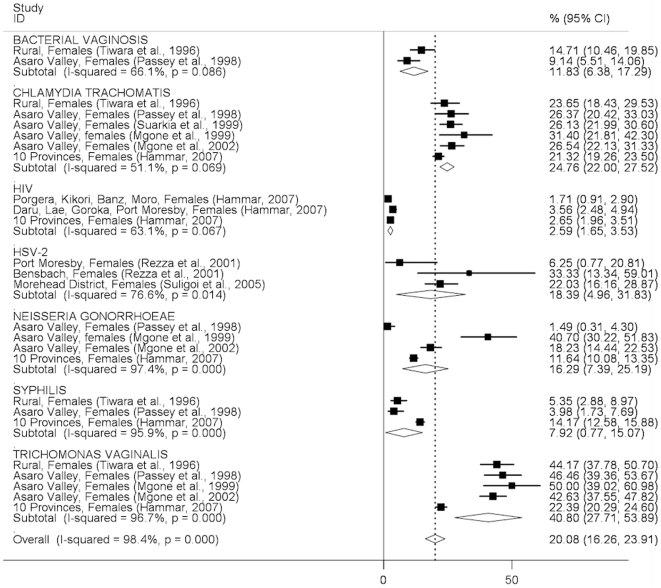
Prevalence of sexually transmitted infections in women: community-based studies. Forest plots showing unadjusted prevalence estimates (boxes) with 95% confidence limits (bars). Pooled prevalence estimates are represented as diamonds in this plot.

**Figure 4:**
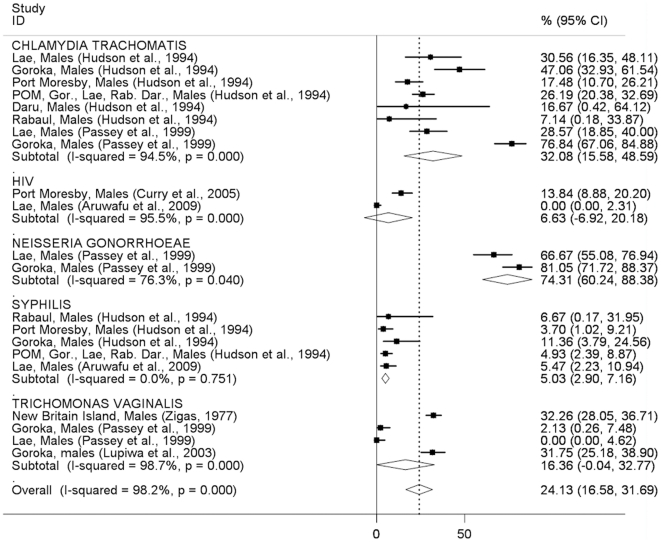
Prevalence of sexually transmitted infections in men: clinic-based studies. Forest plot showing unadjusted prevalence estimates (boxes) with 95% confidence limits (bars). Pooled prevalence estimates are represented as diamonds in this plot.

**Figure 5:**
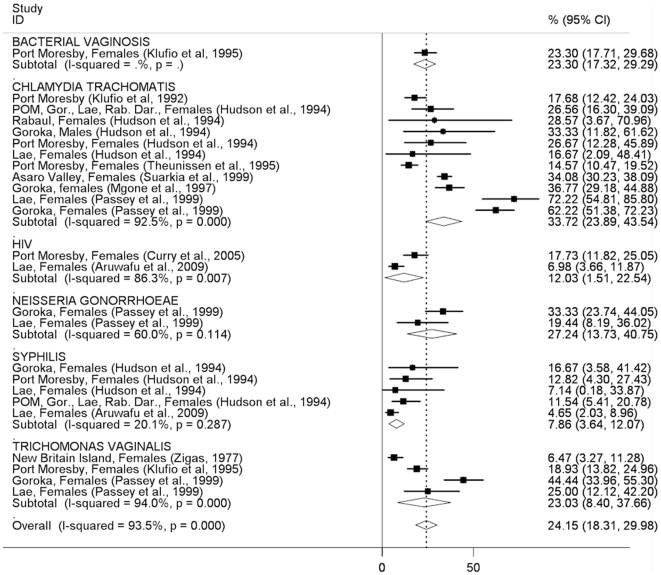
Prevalence of sexually transmitted infections in women: clinic-based studies. Forest plot showing unadjusted prevalence estimates (boxes) with 95% confidence limits (bars). Pooled prevalence estimates are represented as diamonds in this plot.

**Figure 6:**
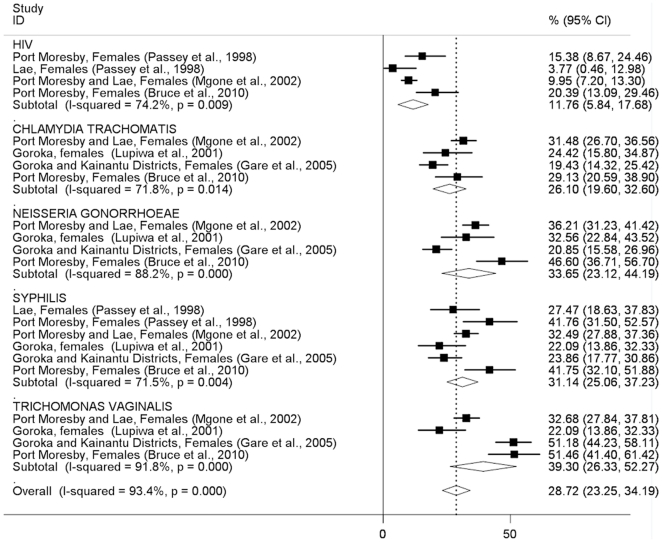
Prevalence of sexually transmitted infections among female sex workers. Forest plot showing unadjusted prevalence estimates (boxes) with 95% confidence limits (bars). Pooled prevalence estimates are represented as diamonds in this plot.

In general, the pattern of STI prevalence among both men and women indicates substantially higher prevalences in clinic-based than community-based studies, with some notable exceptions (syphilis in men, trichomonas in women; Figures 2–5). The prevalence of STIs among FSWs was typically higher than among women or men participating in community-based studies conducted in the general population (Figure 6), although estimated prevalences of chlamydia were comparable.

The pooled *Chlamydia trachomatis* prevalence was similar among men (20.2%; 95%CI:11.5–28.9%) and women (24.8%; 95%CI:22.0–27.5) in community-based studies (Figures 2, 3) and was around 30% among both men and women in clinic-based studies (Figures 4, 5). The pooled prevalence estimate among FSWs was 26.1% (95%CI:19.6–32.6) (Figure 6).

The pooled prevalence estimate of gonorrhoea in community-based studies was 10.0% (95%CI:8.2–11.8) in men and 16.3% (95% CI 7.4–25.2) in women. A single clinic-based study (Passey et al, 1999) conducted in Goroka and Lae provided a pooled prevalence estimate of 74.3% (95%CI:60.2–88.4) among men and 27.2% (95%CI:13.7–40.8) in women but was specifically designed to identify cases of urethral or vaginal discharge in order to study gonococcal antibiotic sensitivity and hence is difficult to interpret. Among FSWs, the overall prevalence of gonorrhoea was estimated at 33.7% (95%CI:23.1–44.2), based on four published studies.

The pooled prevalence estimate of syphilis in community-based studies was 12.9% (95%CI:11.2–14.5) in men and 7.9% (95%CI:0.8–15.1) in women. In men, community-based estimates were higher than those reported in clinic-based studies (5.0%; 95% CI:2.9–7.2). In women, clinic-based estimates (7.9%, 95%CI:3.6–12.1) were comparable to community estimates. The pooled prevalence estimate among FSWs was 31.1% (95%CI:25.1–37.2).

The prevalence of *Trichomonas vaginalis* was similar in community and clinic-based studies in men (12.3%; 95%CI:10.2–14.3 *vs.* 16.4%; 95%CI:0.0–32.8) but higher in community-based studies compared to clinic-based studies in women (40.8%; 95%CI:27.7–54.0 *vs.* 23.0%; 95%CI:8.4–37.7). The pooled prevalence estimate among FSWs was comparable to that observed in community-based studies among women in the general population (39.3%; 95%CI:26.3–52.3).

HSV-2 pooled prevalence in community-based studies was an estimated 22.8% (95%CI:4.9%–40.6%) in men (ranging from 8.2% to 33.1% in individual studies), and 18.4% (95%CI:5.0–31.8%) in women (ranging from 6.3% to 59.0%). There were no prevalence estimates from clinic-based or female sex worker studies.

HIV prevalence in community-based studies was 1.8% (95%CI:1.2–2.4) in men and 2.6% (95%CI:1.7–3.5) in women but substantially higher among FSWs (11.8%; 95%CI:5.8–17.7). A study of patients attending the Emergency Department at Port Moresby General Hospital reported surprisingly high HIV prevalence estimates:13.8% in men and 17.7% in women[47].

Among women in the general population, the prevalence of bacterial vaginosis was 11.8% (95%CI:6.4–17.3) in community-based studies and 23.3% (95%CI:17.3–29.3) in a single clinic-based study from Port Moresby. No estimates were available among FSWs.

Six studies reported age-specific prevalences [26], [32], [38], [47], [49], [50] but data were not reported in sufficient detail to enable summary age-specific prevalences to be calculated. In men, age-specific prevalence was reported for HSV-2 only[49]. In women, chlamydia was most prevalent in younger women in both the general population where prevalences of around 40–45% were observed among women aged <25 y in rural Eastern Highlands[32], [38]; and among FSWs <25 y, where similar prevalences were reported in the same province[26]. The prevalence of gonorrhoea was higher in younger (<25 y) FSWs[26]; no trend by age was observed among women in the wider community[32]. The prevalence of trichomonas was high and similar across age groups among both rural women and FSWs[26], [32], [38]. In both men and women, HSV-2 sero-prevalence increased with age[49], [50]. A single study reported age-specific HIV prevalence, but found no significant trend by age[47].

Important differences were observed between urban and rural prevalence estimates for several STIs, most notably HSV-2 (two estimates among men in rural areas of 27.8%[49] and 33.1%[50], compared to 8.2%[49] in Port Moresby, with similar differentials observed among women in the same studies).

## Discussion

This review illustrates the complexity, diversity and heterogeneity of the epidemiology of STIs and HIV among men and women in PNG according to geographical location, urban/rural setting and by apparent *a priori* sexual risk category. The prevalence of chlamydia and trichomonas appear similar in community-based general population surveys compared to so-called high-risk groups such as STI clinic attendees or FSWs, calling into question the validity of simple risk categorisation in this setting. Prevalences of gonorrhoea, syphilis and HIV were however much higher among such at-risk groups and the likelihood of significant selection bias in the study by Passey et al (1999)[35], which reported the highest clinic-based prevalence estimates of chlamydia and gonorrhoea in both men and women in the review, suggests that comparisons between sub-populations be made with caution, particularly where the only available estimates are from a single or very limited number of studies.

High prevalences of HIV, sexually transmitted and genital infections were observed among men and women in PNG compared to many other countries in the Asia-Pacific region. In a recent six country study among 1678 pregnant women attending urban and rural antenatal clinics in Fiji, Kiribati, Samoa, Solomon Islands, Tonga and Vanuatu, the prevalence of chlamydia was 6.4–29.0% (mean 18.0%); gonorrhoea, 0.0–2.5% (mean 1.7%); and syphilis, 0.0–10.0% (mean 3.0%) in the period 2004–2005[57]. None of the 1618/1678 clinic attenders who underwent voluntary counselling and confidential HIV testing (VCT) were HIV sero-positive[57], [58]. Chlamydia was the most prevalent STI, particularly in Fiji (29.0%) and Samoa (26.8%) where rates were comparable to those observed among women in PNG. As in PNG, chlamydia was more prevalent in younger women in all locations. For example, in Tonga, prevalence among women <25 y was 27.5%, compared to 8.3% among women >25 y; in Samoa prevalences of 40.7% and 17.5% were observed in these age-groups respectively[58]. Only 1.5% of the 1678 antenatal clinic attenders reported transactional or commercial sex in the previous 12-months, but these women were six times more likely to have chlamydia infection[58].

High rates of STIs among pregnant women, including chlamydia (21.5%), gonorrhoea (5.9%), HSV-2 (30.0%), syphilis (2.4%) and trichomonas (27.5%), have also been reported previously in Vanuatu[59], [60], [61] and similar rates observed in Fiji[62] and Samoa[63], but estimates from other Pacific Island nations are unavailable. Differences in STI prevalence between urban and rural settings observed in PNG have also been reported in other Pacific Island countries, for example trichomoniasis among non-pregnant women in rural communities in Ambae Island (43.4%) was more common than among urban antenatal clinic attenders in Port Vila (14.7%)[64]. Data from community and clinic-based surveys on HIV/STI prevalences in men, non-pregnant women and FSWs are currently available in PNG only.

HIV/STI prevalences among transactional and commercial sex workers in PNG were high in this review, and broadly comparable to those observed among similar populations in Indonesia[65], [66], [67], [68], the Philippines[69], [70] and other countries in SE Asia[71], [72], [73], [74], [75]. Prevalences among men and women in the general community however appear much higher in PNG (and in several other Pacific Island nations, such Fiji, Samoa and Vanuatu) than in SE Asia [76], [77]. For example, among 451 antenatal clinic attenders in Cambodia, the prevalence of chlamydia was 2.8%; gonorrhoea, 0.0%; syphilis, 1.3%; and trichomonas 2.7%[77]. In a general population survey among 2550 women and 1350 men in the Philippines, the prevalence of chlamydia was 5.7%, gonorrhoea 0.8%, and syphilis 0.2% among women; and 4.4%, 1.1% and 0.2% among men, respectively[76]. The reasons for these differences are unclear, but felt likely to be the result of locally-specific interactions between the behavioural determinants, socio-cultural dimensions and structural contexts that frame sexual agency, sexuality and sexual health in PNG[9], [11], [78], [79], [80], [81], [82], [83], [84], [85]. These include gender power disparities, sexual violence and the societal roles of men and women[9], [11], [78], [79], [80], [83], [85], [86]; low levels of male and female condom use[27], [33], [38], and of male circumcision[11], [86], [87], [88]; limited access to STI treatment services due to poor transport and health systems infrastructure[4], [89]; and limited success in the design and implementation of culturally-relevant behaviour change interventions among both general population and at-risk groups, such as truck drivers, male and female sex workers and their clients[8], [11], [88]. These factors may also explain the high HIV/STI prevalences observed in Tanah Papua Province in Eastern Indonesia, which is experiencing a generalised HIV epidemic that has many parallels to that of neighbouring PNG[90]. HIV prevalence among men and women aged 15–49 y in Tanah Papua was recently estimated at 2.4%, the highest in Indonesia and approximately 15 times the national average. Among ethnic Papuans, HIV prevalence was more than twice that of non-ethnic Tanah Papua residents (2.8%, 1.5% respectively).

How much confidence can be placed in the findings of this review, given the available evidence? We included only studies that reported laboratory-confirmed estimates and excluded those providing clinical diagnoses alone, removing a potential source of misclassification bias. Laboratory diagnostic algorithms changed considerably over the study period, particularly from the mid-1990s onwards when highly-sensitive single and multiplex PCR assays began to replace highly-specific wet mount and Gram stain microscopy and bacteriological culture as primary diagnostic tools for gonorrhoea and trichomonas (Table S1). The re-testing by Mgone et al (2002)[32] of stored genital specimens originally tested bacteriologically for *N. gonorrhoeae* in an earlier community-based study[38] resulted in an increase in estimated prevalence in women from 1.5% (95%CI:0.3–4.3) to 18.2% (95%CI:14.4–22.5) (Figure 3). Direct comparisons of diagnostic test performance are not possible for other studies in this review that used different assays at different time points, suggesting that pooled STI prevalence estimates be interpreted with caution, particularly those which include studies conducted over a wide time period (such as chlamydia in men, Figure 2; or gonorrhoea in women, Figure 3). Specimen collection method may also have affected prevalence estimates: all studies among FSWs that reported chlamydia, gonorrhoea and trichomonas used self-collected vaginal swabs, in contrast to community and clinic-based studies in which swabs were collected by a trained clinician. It is possible that this may have affected prevalence estimates among FSWs due to differences in specimen quality and site of collection (e.g. endocervix *vs.* vaginal wall or introitus)[91], [92], resulting in lower estimated prevalences of chlamydia and gonorrhoea in sex workers compared to other women, but likely to have had less effect on trichomonas estimates; a pattern reflected in the results of this review (Figures 3, 5, 6). The prevalence of syphilis may have been overestimated in many sub-populations because RPR, VDRL and TPHA assays are unable to distinguish *T. pallidum* from *T. pertenue* infection, the cause of yaws, which has remained endemic in many parts of PNG despite the eradication efforts of the 1950's and 1960's[93], [94], [95], [96].

Selection bias may have led to an over or under estimation of STI and HIV prevalence in different sub-populations. The majority of community-based studies used random population-based sampling[32], [37], [38], although a recent multi-site study used convenience sampling in randomly selected areas[27]. The extent of selection bias in these studies cannot be ascertained as participation rates were not reported. One study reported analysing separately those participants who volunteered but were not included in the random sample[37], [38]. Men and women who ‘self-selected’ for inclusion in community-based studies (as in the study by Tiwara et al[37]) may have been more likely to have genital symptoms than those who were randomly selected to participate. Studies among FSWs may have been similarly biased or alternatively, failed to reach the most vulnerable women at greatest risk of HIV/STIs (e.g. young women with limited ability to negotiate condom use or clinic attendance with their spouse/sexual partners). All studies used self-identification in their definition of female sex worker, which may have excluded some women who engage in commercial and/or transactional sex but who do not identify themselves in this way (e.g. bar workers[86]). The lack of HIV/STI prevalence estimates among MSM populations in PNG is cause for concern[2], [8].

Due to the limited data available it was not possible to construct robust time trends in HIV/STI prevalences in this review. The pooled STI/HIV prevalence estimates presented were each derived from studies conducted over several decades, suggesting they be interpreted with caution.

Publication bias was unlikely to have been significant in this review, as both published and unpublished data were located. Our study included all peer-reviewed articles located through systematic searches of medical databases (including secondary searching of bibliographies), but also included manual searches of conference abstracts and other reports held at the PNGIMR Medical Library to locate studies that do not currently appear in the peer-reviewed literature. The review team includes senior public health professionals from the National Department of Health and the National AIDS Council in PNG, many of whom have worked in this field since the 1980s and were able to identify and locate data that would otherwise have been unavailable for this systematic review.

The findings of this systematic review have a number of important implications. First, it suggests a re-appraisal of priorities and systems for HIV/STI surveillance in PNG to enable more robust, valid and reliable prevalence estimates to be generated. A renewed focus on the systematic generation and synthesis of routine data collected at multiple sentinel surveillance sites, established at designated antenatal and STI clinics throughout PNG, is needed and could be supplemented and validated by periodic (e.g. every 3–5 y) national integrated bio-behavioural surveys[8], [45], [88], [90]. Second, information from routine and periodic surveillance could be further validated and strengthened by highly-focussed, policy-relevant research to address key knowledge gaps in the epidemiological and socio-behavioural profile of specific sub-populations, particularly those in rural areas and those considered at high-risk of infection, for example by establishing longitudinal clinical and qualitative cohort studies[8]. Third, this review highlights our limited knowledge of the epidemiology of a number of STIs in PNG, particularly HSV-2, thought to be a key co-factor in many generalised HIV epidemics[17] and for which no clinic-based prevalences or estimates among FSWs are currently available. A more accurate understanding of these issues will be key to developing effective, locally-appropriate behaviour change communication and biomedical prevention strategies for improved sexual health in PNG.

## Supporting Information

Table S1 Studies reporting prevalences of HIV, STIs and genital infections in community and clinic-based settings and among female sex workers in Papua New Guinea, 1950–2010^§^.
(DOCX)
