# Supplementary material for: The Prevalence of Sexually Transmitted Infections in Papua New Guinea: A Systematic Review and Meta-Analysis
Source: PLoS One. 2010 Dec 23;5(12):e15586. doi: 10.1371/journal.pone.0015586 (PMC3009733; doi:10.1371/journal.pone.0015586)
Supplement: Table S1 — Studies reporting prevalences of HIV, STIs and genital infections in community and clinic-based settings and among female sex workers in Papua New Guinea, 1950–2010§. (DOCX) [file pone.0015586.s001.docx]

**Table S1.** Studies reporting prevalences of HIV, STIs and genital infections in community and clinic-based settings and among female sex workers in Papua New Guinea, 1950-2010^§^.

| Author | Year of data collection | Study design / population | Age range | Location | Setting | STI/genital infections | Laboratory diagnostics |
| --- | --- | --- | --- | --- | --- | --- | --- |
| ***a) Community-based studies (n=10)*** | | |  |  |  |  |  |
| Tiwara et al., 1996[37] | 1995 | 201 women and 169 men recruited in stratified random cluster sample of 16 villages; 243 self-selected women and 85 self-selected men also participated and provided specimens | ≥15 y (men); 15-45 y (women) | Asaro Valley, Eastern Highlands Province | Rural | *C. trachomatis* | Nested PCR as per Hayes et al, 1992[97] |
|  |  |  |  |  |  | *N. gonorrhoeae* | Gram stain; gonococcal culture |
|  |  |  |  |  |  | Syphilis | RPR/TPHA (Murex system) |
|  |  |  |  |  |  | *T. vaginalis* | Wet mount for mobile trichomonads |
|  |  |  |  |  |  | Bacterial vaginosis | See below^†^ |
| Passey et al., 1997[42] | 1997 | Random cluster sample of 198 women in a rural area of the highlands | Women of reproductive age | NS | Rural | *T. vaginalis* | NS |
| Passey et al., 1998[38,41] | 1995 | 201 women recruited in stratified random cluster sample of 16 villages | 15-45 y (women) | Asaro Valley, Eastern Highlands Province | Rural | *C. trachomatis* | Nested PCR as per Hayes et al, 1992[97] |
|  |  |  |  |  |  | *N. gonorrhoeae* | Gram stain; gonococcal culture |
|  |  |  |  |  |  | Syphilis | RPR/TPHA (Murex system) |
|  |  |  |  |  |  | *T. vaginalis* | Wet mount for mobile trichomonads |
|  |  |  |  |  |  | Bacterial vaginosis | See below^†^ |
| Mgone et al., 1999[31] | 1995 | 86 stored vaginal swabs collected during earlier community-based surveys | 15-45 y (women) | Asaro Valley, Eastern Highlands Province | Rural | *C. trachomatis* | Multiplex PCR (as per Mahony et al, 1995[98]) for both *C. trachomatis* and *N. gonorrhoeae* on vaginal swabs (previously stored at -80^0^C for >4y). |
|  |  |  |  |  |  | *N. gonorrhoeae* |  |
|  |  |  |  |  |  | *T. vaginalis* | PCR (NS) |
| Suarkia et al., 1999[35] | 1995 | 253 men and 421 women recruited from rural communities | Not reported | Asaro Valley, Eastern Highlands Province | Rural | *C. trachomatis* | Nested PCR as per Hayes et al, 1992[97] |
| Yapea et al., 1999[48] | 1998 | 77 male clients of female sex workers | Adult men | Port Moresby | Urban | HIV | Immuno-Comb II HIV 1& 2 BiSpot (Orgenics, Israel); Serodia-HIV (Fujirebio, Japan); Capillus HIV-1/HIV-2 (Cambridge Diagnostics, Ireland) |
| Rezza et al., 2001[49] | 2001 | 150 participants from 6 remote villages | 15-85 y | Bensbach, Southwest Papua New Guinea | Urban and Rural | HSV-2 | IgG-based type-specific HSV-2 ELISA (Gull Laboratories, Salt Lake City) |
| Mgone et al., 2002[32] | 1995 | Re-testing of specimens collected in an earlier community-based survey[37,38] | 15-45 y (women) | Asaro Valley, Eastern Highlands Province | Rural | *C. trachomatis* | Multiplex PCR (as per Mahony et al, 1995[98]) for both *C. trachomatis* and *N. gonorrhoeae* |
|  |  |  |  |  |  | *N. gonorrhoeae* |  |
|  |  |  |  |  |  | *T. vaginalis* | PCR (as per Riley et al, 1992[99]) |
| Suligoi et al., 2005[50] | 1999,  2001 | 407 participants recruited from 6 remote villages | 15-85 y | Morehead District, Western Province | Rural | HSV-2 | IgG-based type-specific HSV-2 ELISA (Gull Laboratories, Salt Lake City) |
| Hammar, 2007[27] | 2007 | 3407 respondents from 11 field sites in 10 provinces | Males, females  10-59 y | Daru, Lae, Goroka, Port Moresby, Porgera, Kikori, Banz, Moro, Tabubil, Vanimo and Wewak | Urban and rural | *C. trachomatis* | PCR (NS) |
|  |  |  |  |  |  | *N. gonorrhoeae* | PCR (NS) |
|  |  |  |  |  |  | Syphilis | ELISA (NS) |
|  |  |  |  |  |  | *T. vaginalis* | PCR (NS) |
|  |  |  |  |  |  | HIV | ELISA (NS) |
| ***b) Clinic-based studies (n=10)*** | | |  |  |  |  |  |
| Zigas, 1977 [44] | 1977 | 379 men and women attending Health Centres and 259 Caucasian men and women attending outpatient clinics and private practitioners | ≥ 15 y | New Britain Island | Rural | *T. vaginalis* | Diluted May-Gruenwald and Giemsa stain, examined under oil immersion lens |
| Hudson et al., 1994[28] | 1994 | 340 new patients (men and women) attending STI Clinics in five towns | Not reported | Port Moresby, Goroka, Lae, Rabaul, Daru | Urban and rural | *C. trachomatis* | Direct immunofluorescence (MicroTrak, Syva, California) |
|  |  |  |  |  |  | *N. gonorrhoeae* | Gram stain, gonococcal culture^ψ^ |
|  |  |  |  |  |  | Syphilis | VDRL/TPHA/FTA-Abs |
|  |  |  |  |  |  | Donovanosis | Giemsa stained smear from genital ulcer swab |
| Suarkia et al., 1999[35] | 1995 | 581 women presenting in labour to Goroka Base Hospital | Not reported | Goroka | Urban and rural | *C. trachomatis* | Nested PCR as per Hayes et al, 1992[97] |
| Theunissen et al., 1995 [36] | 1995 | 254 women attending Obstetrics and Gyneacology Dept, Port Moresby General Hospital | 17-43 y | Port Moresby | Urban | *C. trachomatis* | Direct immunofluorescence (MicroTrak, Syva, California)  PCR (as per Claas et al., 1991[100]) |
| Klufio et al., 1995[43] | 1995 | 206 pregnant women attending first antenatal clinic visit at Port Moresby General Hospital | Not reported | Port Moresby | Urban | *T. vaginalis* | Bacteriologically confirmed diagnoses (NS) |
|  |  |  |  |  |  | Bacterial vaginosis | Bacteriologically confirmed diagnoses (NS) |
| Mgone et al., 1997[30] | 1997 | 155 women in labour attending Goroka Hospital | Not reported | Goroka | Urban and rural | *C. trachomatis* | Direct flourescent antibody staining (NS) and PCR (NS) |
| Passey et al., 1999[34] | 1997 | 299 new patients attending Goroka and Lae STI Clinics | Male, females  15-65 y | Goroka and Lae | Urban | *C. trachomatis* | Nested PCR as per Hayes et al, 1992[97] |
|  |  |  |  |  |  | *N. gonorrhoeae* | Gram stain, gonococcal culture |
|  |  |  |  |  |  | *T. vaginalis* | In-Pouch (Biomed Diagnostics,USA) |
| Lupiwa et al., 2003[40] | 1997 | Re-testing of stored specimens collected from 189 male STI Clinic attendees | Not reported | Goroka | Urban | *T. vaginalis* | PCR (as per Riley et al, 1992[99]) on urethral swabs (previously stored at -80^0^C for >4y). |
| Curry et al., 2005[47] | 2003 | 300 patients attending Emergency Dept, Port Moresby General Hospital | Male, females  10-69 y | Port Moresby | Urban | HIV | Serodia-HIV (Fujirebio, Japan); Capillus HIV-1/HIV-2 SR (Trinity Biotech, Ireland); Determine HIV-1/HIV-2 SR (Abbotts Laboratories, Japan) |
| Aruwafu et al. 2009 [45] | 2008 | 300 clients (128 men; 172 women) attending Lae Friends STI clinic | Male, females  15-68 y | Lae | Urban | Syphilis | NS |
|  |  |  |  |  |  | HIV | NS |
| ***c) Studies among female sex workers (n=5)*** | | |  |  |  |  |  |
| Passey et al., 1998 [46] | 1998 | 144 female sex workers; recruitment strategy NS | Not reported | Port Moresby and Lae | Urban | Syphilis | Serology (NS) |
|  |  |  |  |  |  | HIV | Serology (NS) |
| Lupiwa et al., 2001[29] | 2001 | 86 female sex workers recruited following awareness campaigns | Not reported | Goroka and surrounding area | Urban and rural | *C. trachomatis* | PCR (NS) of self-collected vaginal swab |
|  |  |  |  |  |  | *N. gonorrhoeae* | PCR (NS) of self-collected vaginal swab |
|  |  |  |  |  |  | Syphilis | Serology (NS) |
|  |  |  |  |  |  | *T. vaginalis* | PCR (NS) of self-collected vaginal swab |
|  |  |  |  |  |  | HIV | Serology (NS) |

| Mgone et al., 2002[33] | 1995 | 407 self-identified female sex workers recruited through a community outreach program involving peer-educators | 13-50 y | Port Moresby and Lae | Urban | *C. trachomatis* | Multiplex PCR (as per Mahony et al, 1995[98]) of self-collected vaginal swab for *C. trachomatis / N. gonorrhoeae* |
| --- | --- | --- | --- | --- | --- | --- | --- |
|  |  |  |  |  |  | *N. gonorrhoeae* |  |
|  |  |  |  |  |  | Syphilis | VDRL/TPHA |
|  |  |  |  |  |  | *T. vaginalis* | PCR (as per Riley et al, 1992[99]) of self-collected vaginal swab |
|  |  |  |  |  |  | HIV | Vironostika HIV Uni-Form II *plus O* (Organon Teknika, SA); Immuno-Comb II HIV 1& 2 BiSpot (Orgenics, Israel); Serodia-HIV (Fujirebio, Japan); Capillus HIV-1/HIV-2 (Cambridge Diagnostics, Ireland) |
| Gare et al., 2005[26] | 2001 | 211 self-identified female sex workers recruited through STI and HIV/AIDS awareness workshops | 16-59 y | Goroka and Kainantu Districts | Urban | *C. trachomatis* | Multiplex PCR (as per Mahony et al, 1995[98]) of self-collected vaginal swab for *C. trachomatis / N. gonorrhoeae* |
|  |  |  |  |  |  | *N. gonorrhoeae* |  |
|  |  |  |  |  |  | Syphilis | VDRL/TPHA |
|  |  |  |  |  |  | *T. vaginalis* | PCR (as per Riley et al, 1992[99]) of self-collected vaginal swab |
|  |  |  |  |  |  | HIV | Immuno-Comb II HIV 1& 2 BiSpot (Orgenics, Israel); Serodia-HIV (Fujirebio, Japan); Capillus HIV-1/HIV-2 (Cambridge Diagnostics, Ireland) |
| Bruce et al., 2010[39] | 2003 | 143 female sex workers recruited through peer contact at12 sites in Port Moresby | 18-40y | Port Moresby | Urban | *C. trachomatis* | Applied BioSystem GeneAmp PCR System 9700 (Geneworks, Adelaide, Australia) of self-collected vaginal swabs for *C. trachomatis / N. gonorrhoeae / T. vaginalis* |
|  |  |  |  |  |  | *N. gonorrhoeae* |  |
|  |  |  |  |  |  | *T. vaginalis* |  |
|  |  |  |  |  |  | Syphilis | Hexagon Syphilis Diagnostic Kits (Weisebaden, Germany) and  VDRL/TPHA |
|  |  |  |  |  |  | HIV | Hexagon HIV Diagnostic Kits (Weisebaden, Germany) Serodia-HIV (Fujirebio, Japan); Capillus HIV-1/HIV-2 (Cambridge Diagnostics, Ireland) |

**^NOTES^**

**^§^**All genital specimens collected by trained clinical staff unless otherwise stated

**^†^**Bacterial vaginosis confirmed by presence of at least 3/4 following findings: clue cells on wet mount or Gram stain; vaginal pH >4.5; positive amine test; absence of normal flora on Gram stain

^ψ^Gonorrhoea reported only among clients with dual chlamydia / gonorrhoea infection; possible to provide estimate of chlamydia but not gonorrhoea prevalence alone in this study

ELISA: Enzyme -linked immunosorbent assay; FTA-Abs: Fluorescent *T. pallidum* antibody test with absorption; RPR: rapid plasma reagin; TPHA: *T. pallidum* haemagglutination test; TPI: *T. pallidum* immobilisation test; VDRL: Venereal Disease Research Laboratory test; NS: not specified
